# Toll-like receptor 9 agonist enhances anti-tumor immunity and inhibits tumor-associated immunosuppressive cells numbers in a mouse cervical cancer model following recombinant lipoprotein therapy

**DOI:** 10.1186/1476-4598-13-60

**Published:** 2014-03-19

**Authors:** Li-Sheng Chang, Chih-Hsiang Leng, Yi-Chen Yeh, Chiao-Chieh Wu, Hsin-Wei Chen, Hai-Mei Huang, Shih-Jen Liu

**Affiliations:** 1 Institute of Biotechnology and Department of Life Science, National Tsing Hua University, Hsinchu, Taiwan; 2 National Institute of Infectious Diseases and Vaccinology, National Health Research Institutes, No. 35, Keyan Road, Miaoli County, Zhunan Town 35053, Taiwan; 3 Graduate Institute of Immunology, China Medical University, Taichung, Taiwan

**Keywords:** Vaccine, Innate receptor, Immunotherapy, Human papillomavirus

## Abstract

**Background:**

Although cytotoxic T lymphocytes (CTLs) play a major role in eradicating cancer cells during immunotherapy, the cancer-associated immunosuppressive microenvironment often limits the success of such therapies. Therefore, the simultaneous induction of cancer-specific CTLs and reversal of the immunosuppressive tumor microenvironment may be more effectively achieved through a single therapeutic vaccine. A recombinant lipoprotein with intrinsic Toll-like receptor 2 (TLR2) agonist activity containing a mutant form of E7 (E7m) and a bacterial lipid moiety (rlipo-E7m) has been demonstrated to induce robust CTL responses against small tumors. This treatment in combination with other TLR agonists is able to eliminate large tumors.

**Methods:**

Mouse bone marrow-derived dendritic cells (DCs) were employed to determine the synergistic production of pro-inflammatory cytokines upon combination of rlipo-E7m and other TLR agonists. Antigen-specific CTL responses were investigated using immunospots or *in vivo* cytolytic assays after immunization in mice. Mice bearing various tumor sizes were used to evaluate the anti-tumor effects of the formulation. Specific subpopulations of immunosuppressive cells in the tumor infiltrate were quantitatively determined by flow cytometry.

**Results:**

We demonstrate that a TLR9 agonist (unmethylated CpG oligodeoxynucleotide, CpG ODN) enhances CTL responses and eradicates large tumors when combined with rlipo-E7m. Moreover, combined treatment with rlipo-E7m and CpG ODN effectively increases tumor infiltration by CTLs and reduces the numbers of myeloid-derived suppressor cells (MDSCs), tumor-associated macrophages (TAMs) and regulatory T cells (Tregs) in the tumor microenvironment.

**Conclusion:**

These findings suggest that the dramatic anti-tumor effects of the recombinant lipoprotein together with CpG ODN may reflect the amplification of CTL responses and the repression of the immunosuppressive environment. This promising approach could be applied for the development of additional therapeutic cancer vaccines.

## Introduction

Effective cancer immunotherapies should eradicate cancer cells and block the immunosuppression that occurs in cancer microenvironments [1,2]. Although CTLs play a major role in anti-tumor responses, increasing evidence indicates that the induction of cytotoxic effects is necessary, but not sufficient, to control tumor progression [3]. The function of CTLs is affected by systemic and local immunosuppressive environments associated with tumor growth. The lytic activity of CTLs in the tumor microenvironment can be suppressed by myeloid-derived suppressive cells (MDSCs), tumor-associated macrophages (TAMs) and regulatory T cells (Tregs) that surround the tumor [4-7]. Increasing the number of MDSCs generates natural suppressive activity in cancer patients [8] and tumor-bearing mice [9], and systemic accumulation of MDSCs is induced by various factors associated with cancers and several pathological conditions. In addition, CD4^+^CD25^+^ Tregs increase at tumor sites in mice and humans during lung [10], head and neck [11], breast [12] and ovarian cancers [13]. CD4^+^CD25^+^ Treg-depleting approaches have revealed that reduced Treg numbers improve anti-tumor responses and the inhibition of tumor growth [14,15]. Accordingly, successful cancer immunotherapy requires modulation of the immunosuppressive effects of tumor-associated MDSCs, M2 macrophages and Tregs.

Bacterial lipoproteins can be modified at the N-terminus with di- or triacyl glyceryl-cysteine units, which are recognized by TLR2 [16,17]. In addition to their TLR2 activity and their ability to induce dendritic cell maturation, recombinant lipoproteins stimulate a cytokine expression profile that is different from that of synthetic lipopeptides [18]. We recently applied this platform technology to produce a recombinant mutant form of E7 (rlipo-E7m) to treat HPV-associated diseases. We observed that the administration of rlipo-E7m completely inhibited tumor growth [19]. In the present study, we used the TLR9 agonist CpG ODN in combination with rlipo-E7m to treat large tumors. Our data indicate that the combination of rlipo-E7m and CpG ODN dramatically eliminates large tumors. Moreover, CpG ODN synergized with a TLR2 agonist–conjugated antigen to induce the systemic and local production of cytotoxic CD8^+^ T cells and decrease the number of immunosuppressive cells both locally and systemically. These findings suggest that CpG ODN combines with a recombinant lipoprotein exhibiting TLR2 agonist activity to enhance anti-tumor immunity and block local immunosuppressive cells. These results demonstrate that the combination of CpG ODN and recombinant lipoprotein represents a feasible approach for the development of cancer vaccines.

## Results

### Effects of CpG ODN with rlipo-E7m in DC activation and tumor therapy

**Figure 1:**
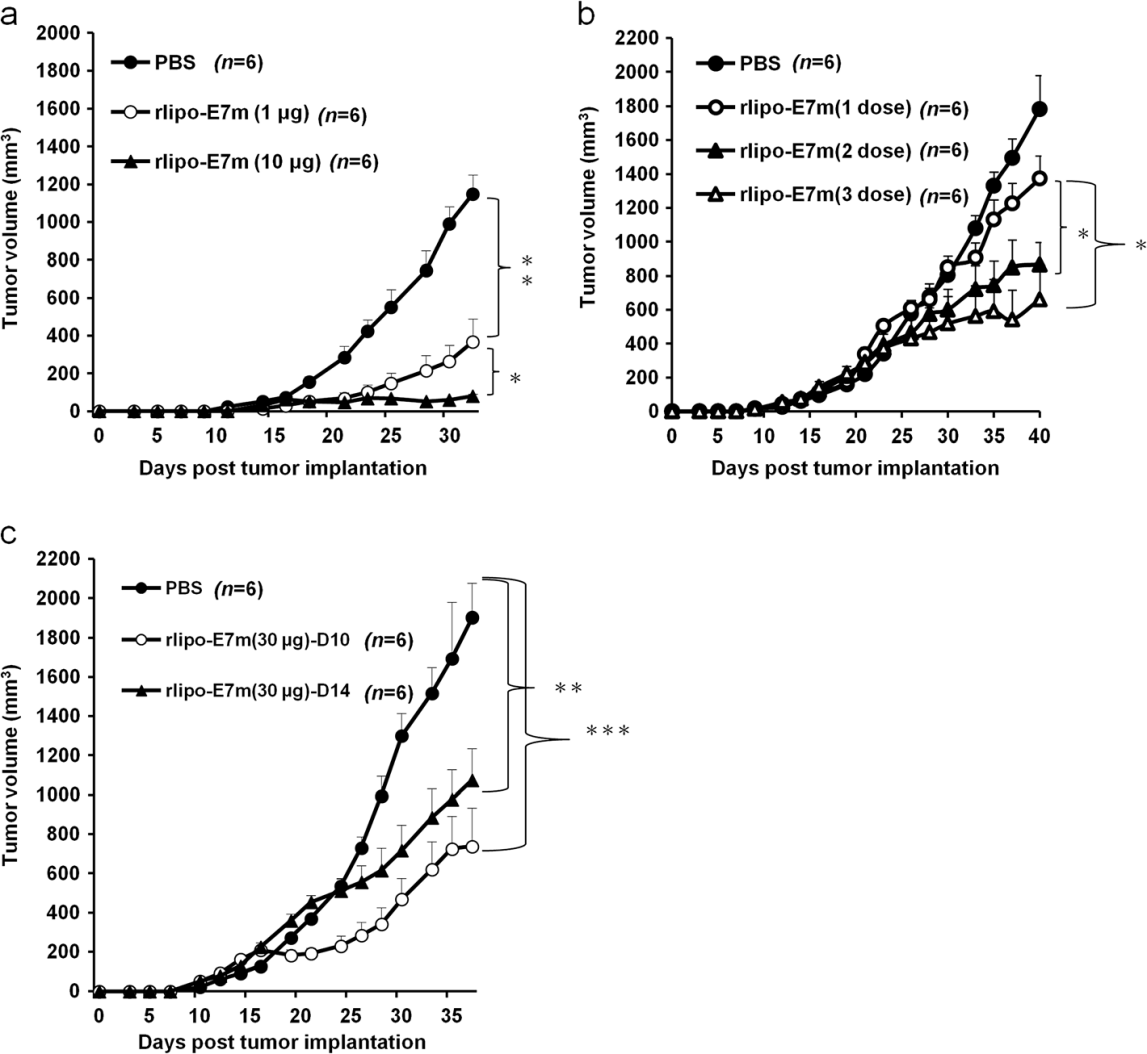
**Anti-tumor effects of rlipo-E7m.** Approximately 2 × 10^5^ TC-1 tumor cells were subcutaneously implanted into C57BL/6 mice. After 7 days, the mice were subcutaneously injected with a single dose of **(a)** PBS, rlipo-E7m (1 μg/mouse) or rlipo-E7m (10 μg/mouse). The tumor-bearing mice were administered 10 μg of rlipo-E7m at 14 days; 14 and 21 days; or 14, 21 and 28 days **(b)**. A single dose of 30 μg of rlipo-E7m was subcutaneously injected into the mice at 10 days or 14 days **(c)**. The tumor volume was calculated using the formula length × width × width/2.

Previously, we demonstrated the ability of rlipo-E7m to induce anti-tumor immunity [19]. However, we observed that a low dose of rlipo-E7m (1 μg) did not inhibit tumor growth (Figure 1a). To examine the therapeutic effects of applying a multiple-dose regimen, tumor-bearing mice were treated with 10 μg of rlipo-E7m with either two (days 14 and 21) or three injections (days 14, 21 and 28). As shown in Figure 1b, multiple doses were ineffective in controlling tumor growth. Moreover, a single-dose injection of 30 μg of rlipo-E7m 10 or 14 days after tumor implantation delayed, but did not completely eradicate, tumor growth (Figure 1c). This result suggests that additional immune-potentiating signals must be incorporated to eliminate large tumors.

**Figure 2:**
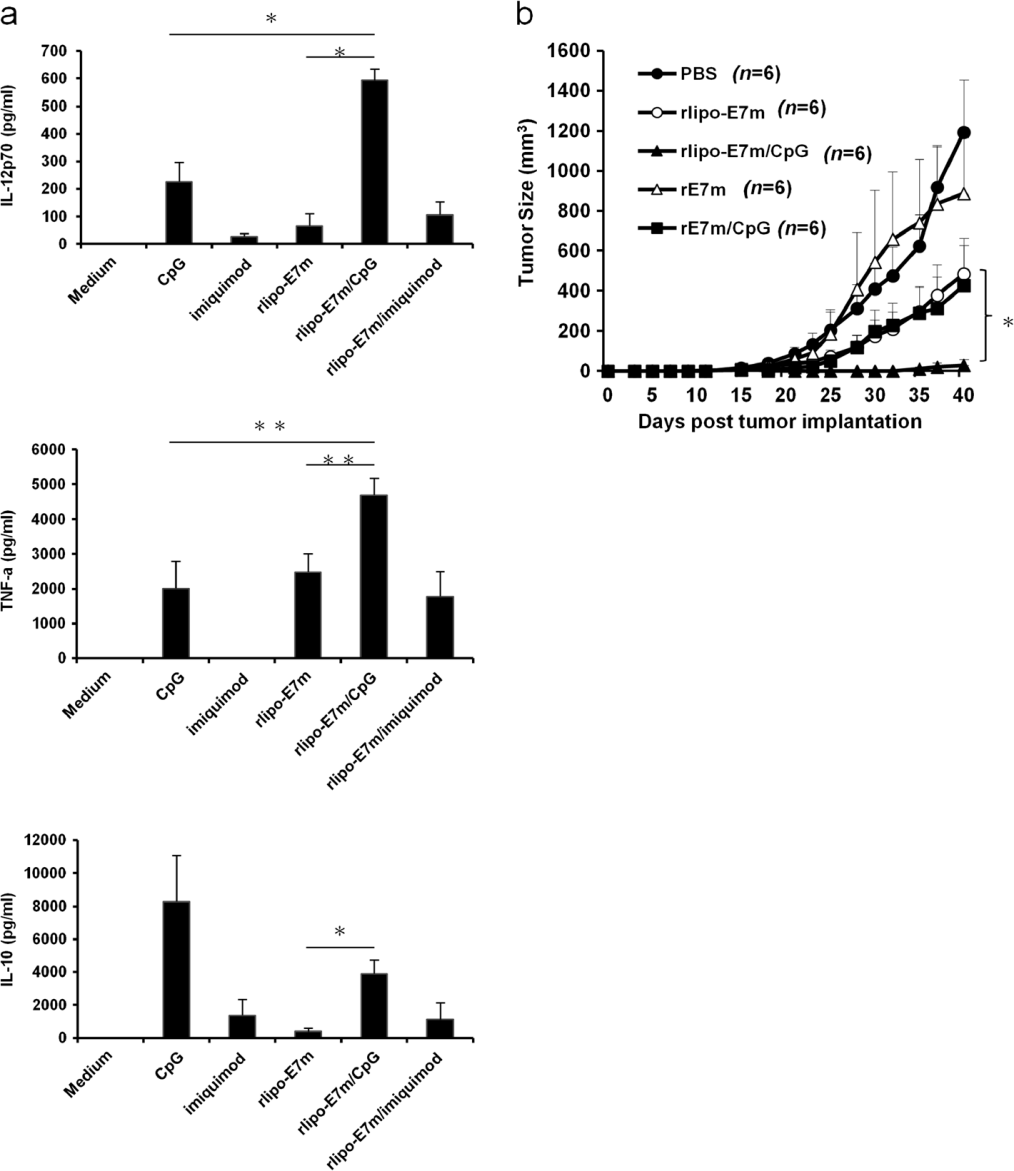
**TLR agonists synergistically enhance rlipo-E7m-induced dendritic cell activation and anti-tumor activity.** Cultured plasmacytoid dendritic cells (pDCs) were incubated with medium, CpG ODN (100 nM), imiquimod (10 μg/ml) or rlipo-E7m (100 nM) ± CpG ODN or imiquimod. The supernatants were collected for the detection of cytokines 24 hours after stimulation. **(a)** Cytokines, including IL-12p70, TNF-α and IL-10, were analyzed by ELISA to assess pDC activation. The data are presented as the means + SD of duplicate DC cultures from three independent experiments. **P < 0.01, ***P < 0.001.**(b)** To evaluate the anti-tumor effect of the combined formulation, tumor-bearing mice were administered a single dose of PBS, rE7m (1 μg/mouse), rE7m (1 μg/mouse) and CpG (10 μg/mouse), rlipo-E7m (1 μg/mouse) or rlipo-E7m (1 μg/mouse) and CpG ODN (10 μg/mouse) via subcutaneous (s.c.) injection 7 days after tumor cell implantation. The tumor volume was calculated using the formula length × width × width/2(mm^3^).

In an attempt to increase DC activation with rlipo-E7m, a TLR7 agonist (imiquimod) and a TLR9 agonist (CpG ODN) were administered to evaluate the synergistic effects of these compounds on plasmacytoid dendritic cells (pDCs). Following stimulation of pDCs, administration of rlipo-E7m combined with CpG ODN (rlipo-E7m/CpG) substantially increased secretion of the pro-inflammatory cytokines IL-12p70 and TNF-α, but no effect was observed when rlipo-E7m was combined with imiquimod (Figure 2a). Interestingly, secretion of the anti-inflammatory cytokine IL-10 following CpG ODN stimulation was reduced under combined treatment with rlipo-E7m (Figure 2a). Based on these results, CpG ODN may be an effective adjuvant for elevating the vaccine efficacy of the recombinant lipoprotein. Subsequently, a single injection of rlipo-E7m/CpG resulted in tumor regression within 40 days of observation (Figure 2b). Moreover, immunization with non-lipidated E7m (rE7m), with or without CpG ODN, was also conducted to determine whether lipidation (i.e., TLR2 stimulation) is indispensable for the observed elevated therapeutic effects. We observed that immunization with rE7m/CpG, but not rE7m alone, delayed tumor growth (Figure 2b). These results suggested that the combination of TLR2 agonist-fused antigen (rlipo-E7m) and the TLR9 agonist (CpG ODN) induced strong anti-tumor effects compared to antigen (rE7m) and CpG ODN. Furthermore, suppression of tumor growth was observed for over 100 days in response to treatment with rlipo-E7m/CpG (Additional file 1: Figure S1a and S1b). These data indicate that rlipo-E7m and CpG ODN induced strong DC activation and the suppression of tumor growth.

### Treatment with rlipo-E7m and CpG ODN enhances antigen-specific T cell immunity

**Figure 3:**
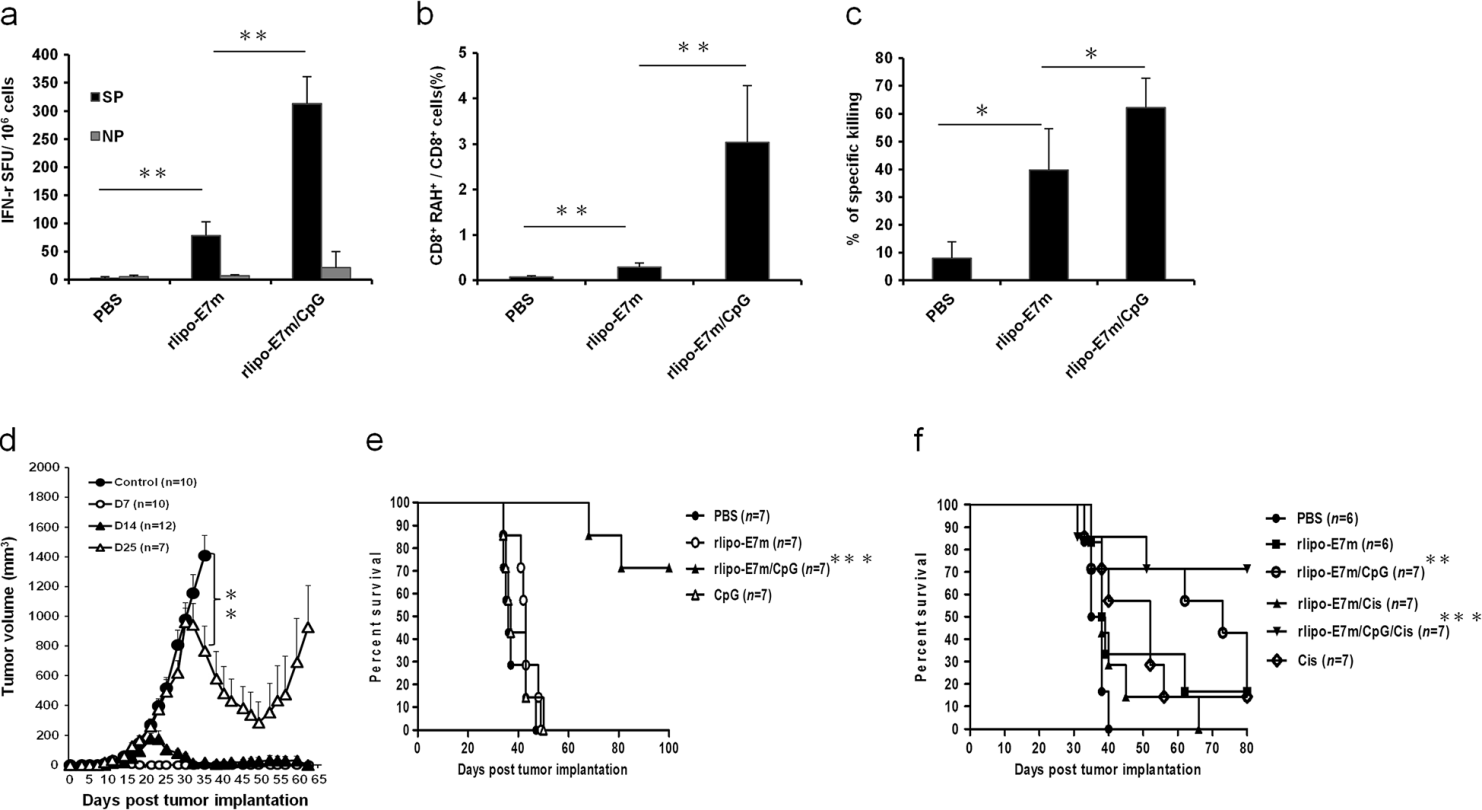
**Immunization with rlipo-E7m and CpG ODN elicits antigen-specific CTL responses and robust anti-tumor effects against large tumors.** Mice were immunized s.c. with rlipo-E7m (10 μg/mouse) with or without CpG (10 μg/mouse) and were boosted once 14 days after the first immunization. The mice were euthanized 7 days after the second immunization. **(a)** The isolated spleen cells were re-stimulated with 10 μg/ml of HPV16E7_49-57_ (RAH)-specific peptide (SP) or non-specific peptide (OVA_257-264_) (NP) for 48 hours, and IFN-γ production was determined using the ELISPOT assay. The data represent the number of IFN-γ spot-forming cells per 10^6^ spleen cells for each duplicate (means + SD). **(b)** The spleen cells were stained with the RAH/MHC I tetramer and anti-CD8 antibody. **(c)** Naïve spleen cells were pulsed with SP or NP and then stained with 5 μM CFSE^hi^ or 0.5 μM CFSE^lo^, respectively. The CFSE-labeled cells were *i.v.* injected into immunized micefor 18 hours, and analyzed by flow cytometry. Specific lysis was determined using the following equation: % specific lysis = [1-(% CFSE^hi^ /% CFSE^lo^)] × 100. **(d)** Tumor-bearing mice were immunized with rlipo-E7m (10 μg/mouse) and CpG (10 μg/mouse) at 7, 14 or 25 days post-TC-1 cell (2 × 10^5^) implantation, and PBS was used as a control. The tumor volume was calculated using the formula length × width × width/2. **(e)** The mice were injected with 2 × 10^5^ TC-1 cells intravenously. After 14 days, the mice were injected with PBS, rlipo-E7m, CpG or rlipo-E7m/CpG subcutaneously. **(f)** Tumor-bearing mice were immunized s.c. with rlipo-E7m in the presence or absence of CpG 25 days following cisplatin (75 μg/mouse) treatment (day 21). Kaplan-Meier analysis was performed on the survival data. (*P < 0.05, **P < 0.01, ***P < 0.001).

To determine whether enhanced CTLs responses of rlipo-E7m *in vivo*, spleen cells from immunized mice were used to induce CTL responses. Following prime and boost immunizations at a 14-day interval, the rlipo-E7m/CpG-mediated induction of E7-specific IFN-γ-secreting cells (312.8 ± 88.98 per 1 million cells) was greater compared to induction with rlipo-E7m alone (78.2 ± 24.6) (Figure 3a). To quantify E7-specific CD8^+^ T cell numbers following immunization, an MHC class I tetramer containing an E7-derived H-2D^b^-restricted CTL epitope was used. The proportion of E7-specific CD8^+^ T cells induced through rlipo-E7m/CpG immunization was higher (3.04% ± 1.24%) than that induced with rlipo-E7m alone (0.29% ± 0.08%) (Figure 3b). To assess the cytolytic effects of these compounds *in vivo*, peptide-pulsed splenocytes were labeled with different concentrations of CFSE and subsequently injected (*i.v.*) into mice. The results indicate that immunization with rlipo-E7m/CpG elicited a higher proportion of killing (62.26% ± 10.41%) than rlipo-E7m immunization (39.8% ± 14.84%) (Figure 3c). Similar results were observed when tumor-bearing mice were immunized with rlipo-E7m/CpG (Additional file 2: Figure S2a). E7-specific CD8^+^ T cell numbers were increased ~60-fold following treatment with rlipo-E7m/CpG compared to treatment with rlipo-E7m alone (Additional file 2: Figure S2b). These results suggest that rlipo-E7m/CpG induced strong CTL responses against these tumors.

### Anti-tumor effects of rlipo-E7m and CpG ODN

Although it was evident that CpG ODN enhanced rlipo-E7m-elicited cellular immunity in tumor-bearing mice, the anti-tumor effects of this treatment against clinically relevant tumors had not been determined. Thus, tumor-bearing mice were treated with rlipo-E7m/CpG on day 7, 14 or 25. The sizes of their tumors were palpable, 0.6-0.8 cm and 1.0-1.2 cm on days 7 and 14 and day 25, respectively. The tumors had completely regressed under treatment with rlipo-E7m/CpG on days 7 and 14 (Figure 3d), and tumor regression was also observed following treatment with rlipo-E7m/CpG on day 25 (Figure 3d). However, growth of the regressed tumors was observed 55 days post-tumor implantation, eventually killing the mice. These findings indicate that rlipo-E7m/CpG could be potentially used to treat large tumors. Furthermore, when the tumor-free mice in the rlipo-E7m/CpG-treated group were rechallenged with TC-1 tumor cells 135 days after tumor implantation, tumor growth was not observed after 100 days (Additional file 3: Figure S3). These data demonstrate that anti-tumor memory is generated by immunization with rlipo-E7m/CpG. To assess the therapeutic effects in a lung metastasis model, cancer cells were inoculated intravenously. A single-dose injection of PBS, rlipo-E7m, CpG or rlipo-E7m/CpG was administered 14 days after tumor inoculation, and only rlipo-E7m/CpG effectively protected the mice. The survival rate 100 days after tumor implantation was 70% in the rlipo-E7m/CpG-treated group (Figure 3e).

Because the large tumor did not completely regress after a single treatment with rlipo-E7m/CpG, a combination with chemotherapy may improve the therapeutic effects. To increase the efficiency of tumor growth inhibition for further immunotherapy, the chemotherapy drug cisplatin was administered four days before immunization. The survival rate of tumor-bearing mice was increased when cisplatin was combined with rlipo-E7m/CpG (Figure 3f). These data further demonstrate that the administration of rlipo-E7m/CpG and cisplatin prolongs the survival time of mice.

To exclude the possibility that this anti-tumor activity reflected non-specific activation of TLR2 (rlipo-E7m) and TLR9 (CpG ODN) in antigen-presenting cells, mice were implanted with another type of tumor cell (EL-4, thymoma) and were treated with rlipo-E7m and CpG ODN. Anti-tumor effects were not observed in the EL-4 tumor-bearing mice (Additional file 4: Figure S4). Taken together, these results suggest that antigen-specific anti-tumor immunity associated with rlipo-E7m is substantially enhanced by CpG ODN.

### Anti-tumor effects of rlipo-E7m and CpG ODN depend on CD8^+^ T cells and TLR9

**Figure 4:**
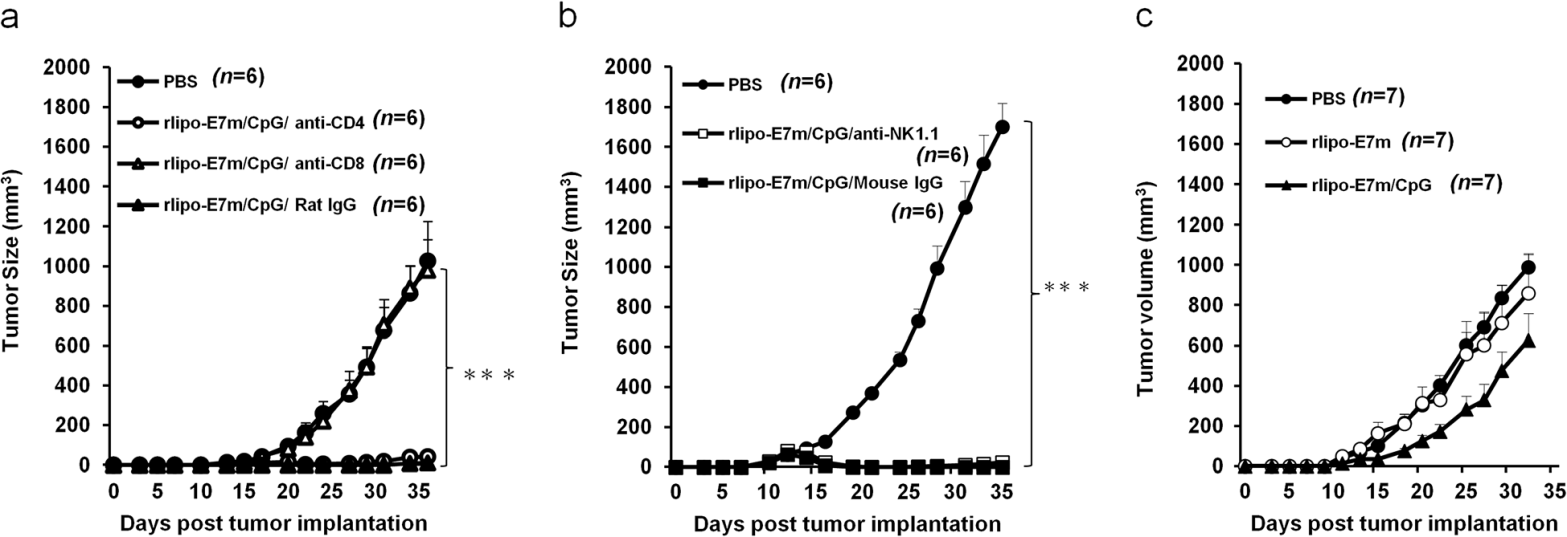
**Anti-tumor effects of rlipo-E7m/CpG are mediated by CD8**^**+ **^**T cells and TLR9 signaling.** C57BL/6 mice (n = 6 per group) were implanted s.c. with 2 × 10^5^ TC-1 tumor cells. Tumor-bearing mice were immunized with PBS or rlipo-E7m/CpG (10 μg/mouse) s.c. 14 days post-tumor cell implantation. **(a)** Mice were i.p. injected with 0.5 mg of anti-CD4, anti-CD8 or rat IgG one day before immunization. **(b)** Mice were i.p. injected with anti-NK1.1 antibody or mouse IgG one day before immunization. **(c)** TLR9-KO mice were implanted s.c. with 2 × 10^5^ TC-1 tumor cells. Tumor-bearing mice were immunized with rlipo-E7m (1 μg/mouse) and CpG (10 μg/mouse) via s.c. injection day 7 post-TC-1 cell implantation. The data show the individual tumor volume after tumor cell implantation. The numbers of mice for each group are indicated in each graph. Tumor volume was calculated by the formula: length × width × width/2(mm^3^).

Although rlipo-E7m alone induces CD8-dependent anti-tumor effects, these effects may differ in the presence of CpG ODN [19]. We treated tumor-bearing mice (7 days) with single i.p. injections of anti-CD8, anti-CD4 or control antibodies (rat IgG) before immunization with rlipo-E7m/CpG. Tumor regression was observed in mice treated with anti-CD4 or rat IgG (Figure 4a). In contrast, the anti-tumor effects were blocked by treatment with anti-CD8. The tumor growth in mice treated with anti-CD8 was similar to the growth observed in mice that were not treated with rlipo-E7m/CpG (Figure 4a). Furthermore, NK cells can be activated or recruited for priming CTL responses by CpG ODN [20]. As seen in Figure 4b, the depletion of NK cells did not block the anti-tumor effects of rlipo-E7m/CpG, and tumor regression was similar to what was observed in the mice treated with control mouse IgG antibody. These data suggest that CpG ODN increased the CD8-mediated anti-tumor effects of rlipo-E7m. To clarify whether TLR9 is necessary for the anti-tumor effects of rlipo-E7m/CpG, tumor-bearing TLR9-knockout mice were treated with PBS, rlipo-E7m or rlipo-E7/CpG. The anti-tumor effects of rlipo-E7m/CpG were lost in the TLR9-knockout mice (Figure 4c). These results demonstrate that the anti-tumor effects of rlipo-E7m plus CpG ODN were the result of contributions from both CD8^+^ T cells and TLR9 signaling.

### The combination of rlipo-E7m and CpG ODN suppresses immune regulators in tumor-bearing mice

**Figure 5:**
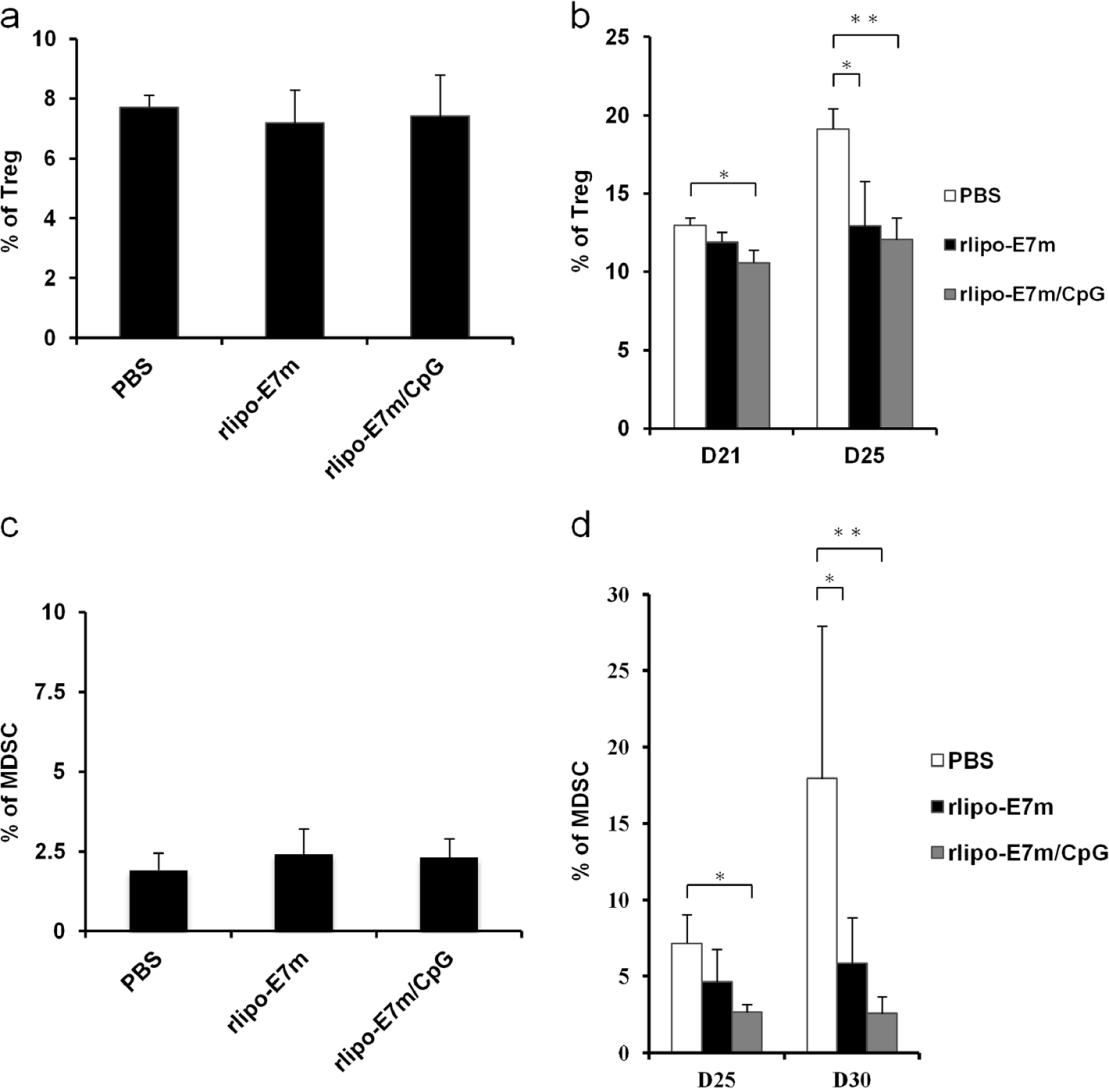
**The combination of rlipo-E7m and CpG leads to the suppression of systemic immunosuppressive cells in tumor-bearing mice.** TC-1 tumor-bearing mice (n = 3 per group) or naïve mice (n = 6 per group) were immunized with rlipo-E7m (10 μg/mouse), rlipo-E7m (10 μg/mouse) + CpG (10 μg/mouse) or PBS as a control. **(a)** Splenic CD4^+^CD25^+^FoxP3^+^ Treg cells derived from immunized tumor-free mice were quantified by flow cytometry 7 days after immunization, and **(b)** cells from tumor-bearing mice were analyzed 21 and 25 days after tumor implantation. The data represent the percentage of splenic Treg cells and are shown as the means + SD. **(c)** Splenic CD11b^+^Gr-1^+^ (MDSCs) cells derived from immunized tumor-free mice were quantified by flow cytometry 7 days after immunization, and **(d)** the MDSCs from tumor-bearing mice were analyzed 25 and 30 days after tumor implantation. The data represent the percentage of splenic MDSCs and are shown as the means + SD. *P < 0.05, **P < 0.01, ***P < 0.001.

In the last decade, tumor-induced immunosuppressive factors and cells in the tumor microenvironment have been identified as major obstacles for cancer immunotherapy. Accordingly, investigation into the effects of rlipo-E7m/CpG immunization on the inhibition of immunosuppressive cells is of interest. Therefore, we attempted to distinguish the systemic and local immunosuppressive cell populations in mice following treatment with rlipo-E7m or rlipo-E7m/CpG. The administration of rlipo-E7m or rlipo-E7m/CpG did not affect the frequency of splenic Tregs in tumor-free mice (Figure 5a). rlipo-E7m or rlipo-E7m/CpG was administered to tumor-bearing mice 14 days after tumor implantation (size was 0.6-0.8 cm in diameter). At 21 or 25 days after tumor implantation, splenic Tregs (CD4^+^CD25^+^FoxP3^+^) were analyzed. The Treg population was reduced by the administration of rlipo-E7m (~13%) or rlipo-E7m/CpG (~12%) 25 days post-tumor implantation compared to the control group (~19%) (Figure 5b). In addition, the frequency of MDSCs was similar among naïve mice in each group (~2%) (Figure 5c). In tumor-bearing mice, the MDSC population increased from ~2% to 7.2% and 17.9% on days 25 and 30, respectively (Figure 5d). Surprisingly, the frequency of MDSCs was reduced to 4.6% and 5.8% following treatment with rlipo-E7m at days 25 and 30, respectively (Figure 5d). Moreover, the administration of rlipo-E7m/CpG completely suppressed the expansion of splenic MDSCs (2.6% and 2.6% on average by days 25 and 30, respectively; Figure 5d). These results indicate that immunization with rlipo-E7m or rlipo-E7/CpG systemically reduces the frequency of both Tregs and MDSCs in tumor-bearing mice.

**Figure 6:**
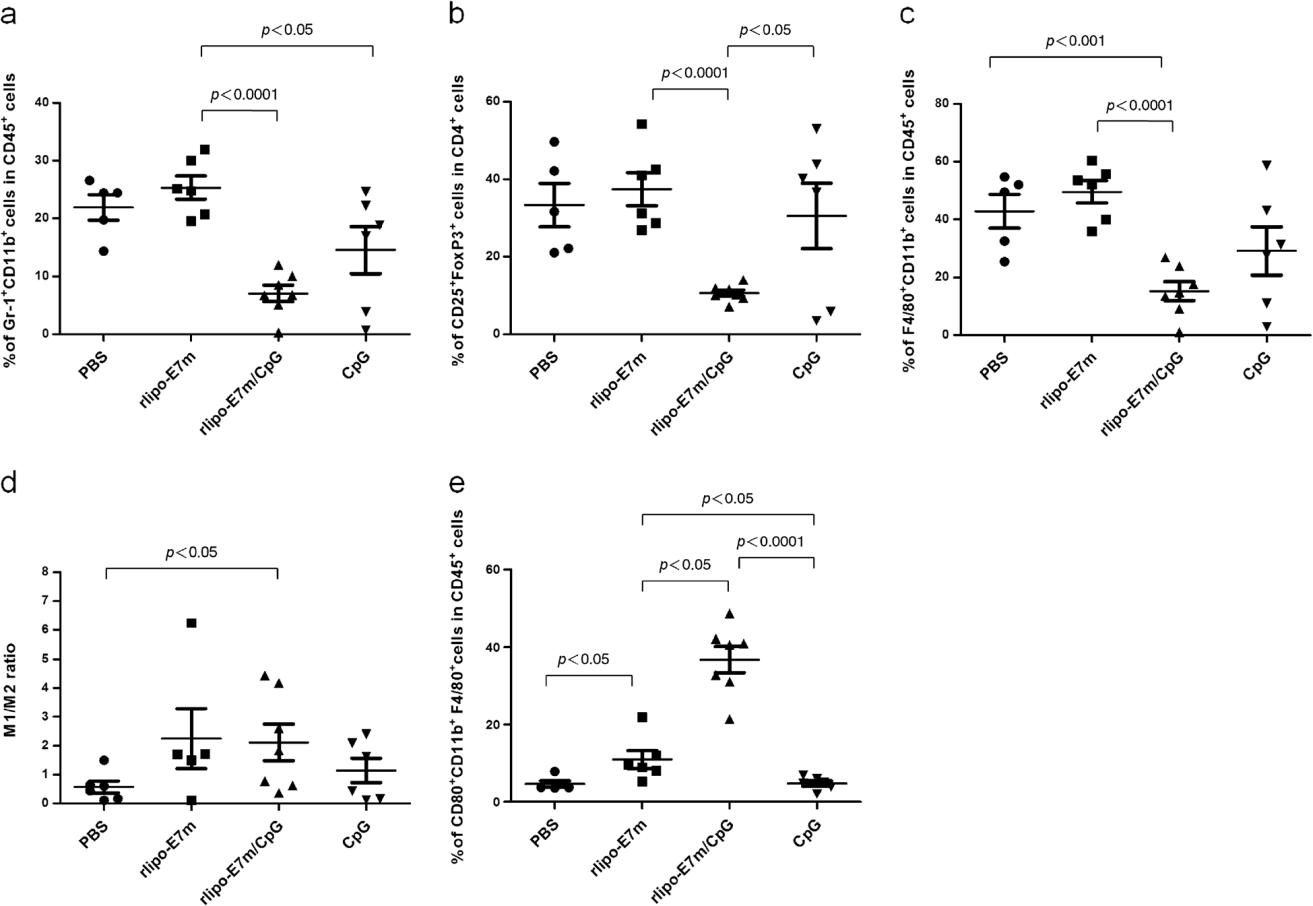
**The combination of rlipo-E7m and CpG leads to the suppression of immunosuppressive cells in the local tumor environment.** Tumor-bearing mice (n = 6 per group) were immunized with rlipo-E7m (10 μg/mouse), rlipo-E7m (10 μg/mouse) + CpG (10 μg/mouse) or PBS as a control 14 days post-tumor cell implantation. The tumors removed from the tumor-bearing mice 24 days post-implantation were minced and crushed through a 70-μm filter, and the total cells were stained with antibodies against the indicated markers; 50,000 events were acquired for each sample. The data represent the percentage of the indicated markers for individual animals. Comparison of the **(a)** MDSC (Gr-1^+^ CD11b^+^), **(b)** Treg (CD4^+^ CD25^+^ FoxP3^+^), **(c)** TAM (F4/80^+^ CD11b^+^), **(d)** TNF-α^+^ TAM/IL-10^+^ TAM and **(e)** CD80^+^ TAM cell populations. Significant differences are indicated by the *P* values in the graph.

To further verify the frequencies of immunosuppressive cells in the tumor microenvironment, tumor tissues were processed and stained for markers of MDSCs, Tregs and TAMs. As shown in Figure 6a, the frequency of tumor-infiltrating MDSCs was suppressed by treatment with rlipo-E7m/CpG (7.06% ± 3.76%) but not by treatment with rlipo-E7m (25.33% ± 4.91%) or CpG ODN alone (Figure 6a). Accordingly, the number of Tregs was also considerably reduced by rlipo-E7m/CpG (10.67% ± 2.17%) treatment but not by treatment with rlipo-E7m (37.41% ± 10.44%) or CpG (30.54% ± 20.69%) compared to the control (33.32% ± 12.49%) (Figure 6b). Interestingly, the TAM population was reduced following treatment with rlipo-E7m/CpG (15.22% ± 8.7%) or CpG (29.1% ± 20.42%) but not after treatment with rlipo-E7m alone (49.56% ± 9.58%) (Figure 6c). Because TAMs can be divided into the inflammatory M1 type (TNF-α^+^/IL-12^+^/iNOS^+^/CD80^+^) and the immunosuppressive M2 type (IL-10^+^/TGF-β^+^Arginase I^+^) [21], the M1/M2 TAM ratio was calculated. The M1/M2 ratio was increased when tumor-bearing mice were treated with rlipo-E7m/CpG but not with rlipo-E7m or CpG alone (Figure 6d). The concentration of the co-stimulatory molecule CD80 was significantly up-regulated on TAMs following treatment with rlipo-E7m or rlipo-E7m/CpG but not after treatment with CpG alone (Figure 6e). These findings imply that rlipo-E7m/CpG increases the number of M1-like TAMs. These data also demonstrate that rlipo-E7m reduces the numbers of immunoregulatory cells in circulation and in the tumor microenvironment in the presence or absence of CpG ODN.

## Discussion

The limited success of cancer immunotherapy reflects the induction of CTL responses that cannot eliminate cancer cells in the presence of tumor-infiltrated immunosuppressive cells (i.e., MDSCs, Tregs and M2 macrophages). To develop a new generation of immunotherapeutic approaches, the enhancement of CTL responses and reduction of immunosuppressive cell numbers must be induced simultaneously. We recently developed rlipo-E7m, which possesses TLR2 agonist activity and robust CD8-mediated anti-tumor activity against palpable tumors in the absence of exogenous adjuvants [19]. In clinical situations, immunotherapeutic reagents must eliminate large tumors and overcome the immunosuppressive barriers of the tumor environment. In this report, we demonstrate that rlipo-E7m alone could not inhibit the growth of large tumors following a single-dose treatment. Although multiple doses did eliminate tumors in some mice, rlipo-E7m in the presence of CpG ODN dramatically eliminated large tumors. In addition, the numbers of tumor-associated immunosuppressive cells, including MDSCs, Tregs and TAMs (especially M2-like cells), were decreased following treatment with rlipo-E7m/CpG. These results demonstrate that rlipo-E7m/CpG not only induces strong CTL responses but also modulates tumor-associated immunosuppressive cells. The synergistic TLR-mediated stimulation of DCs increases the production of inflammatory cytokines and promotes Th1-polarized immunity, which drives CTL responses [22,23]. The combination of TLR3 and TLR9 stimulation enhances the priming efficiency of a DNA vaccine [24]. TLR2/TLR3 agonists or TLR3/TLR9 agonists synergistically activate DCs and subsequently increase the number of activated T cells [25]. These results indicate that high-quality CTL responses are induced through a combination of multiple TLR agonists. TLRs also reduce the immunosuppressive activity of MDSCs [26], TAMs [27,28] and Tregs [29,30].

Although the synergistic activation of DC and T cell responses through multiple TLR agonists has been reported [22,25,31,32], these synergistic effects are not observed when combinations of TLR2 and TLR9 agonists are used [25,32]. Similar results were obtained when rlipo-E7m (a TLR2 agonist) was combined with a TLR9 agonist to stimulate bone marrow-derived DCs (Additional file 5: Figure S5). However, synergistic activation was detected when plasmacytoid DCs were used (Figure 2a). We further measured TLR2 and TLR9 transcripts in different DC subsets and found that the expression of TLR9 was higher in pDCs than in BMDCs or splenic DCs. However, the expression of TLR2 was higher in BMDCs than in pDCs or splenic DCs (Additional file 6: Figure S6). We suggest that the synergistic effects of rlipo-E7m and CpG may be due to the balance of TLR2 and TLR9 expression in pDCs. Moreover, antigen-specific CD8^+^ T cell responses were not improved when the HIV Env-derived peptide (KQIINMWQEVGKAMYAPPISGQIRRIQRGPGRAFVTIGK) was mixed with both TLR2 and TLR9 agonists [32]. In contrast, immunization with the TLR2 agonist-fused antigen (rlipo-E7m) substantially increased CTL responses in the presence of the TLR9 agonist (Figure 3). These conflicting results may result from the lipid moiety of rlipo-E7m, which contains an unsaturated fatty acid, or the fact that rlipo-E7m efficiently co-delivers both antigen and TLR2 agonists to antigen-presenting cells, and the TLR9 agonist might enhance the cross-presentation of antigen to CD8^+^ T cells. We also found that rlipo-OVA/CpG-pulsed pDCs induced higher T cell proliferation than rlipo-OVA alone (Additional file 7: Figure S7a). The rlipo-OVA/CpG-pulsed BMDCs did not induce higher T cell proliferation than rlipo-OVA alone (Additional file 7: Figure S7a). However, it would be nice if the combination could be reproduced in a different antigen model. We produced a model antigen recombinant lipo-ovalbumin (rlipo-OVA) and measured its therapeutic effects with CpG (Additional file 7: Figure S7b). We found that rlipo-OVA/CpG had stronger anti-tumor effects in EG7 (ovalbumin-expressing cell line)-bearing mice than rlipo-OVA alone. These results indicate that TLR9 agonists enhance antigen presentation of TLR2 agonist-fused antigens to CD8^+^ T cells.

In addition to the dramatic anti-tumor effects of rlipo-E7m/CpG, the inhibition of local immunosuppressive cells was also observed following this combined treatment. CpG administration induces differentiation and blocks the immunosuppressive function of MDSCs [26,33] and Tregs [34]. Our results also indicate that the administration of CpG alone mildly reduced the numbers of tumor-infiltrating MDSCs and TAMs. Interestingly, the administration of rlipo-E7m/CpG significantly improved the reduction of immunosuppressive cell numbers compared to treatment with CpG alone (Figure 6). In tumor-bearing mice, a high percentage of M2-like TAMs was detected among tumor-infiltrating leukocytes. This observation is consistent with a previous report indicating that the depletion of TAMs promotes the infiltration of lymphocytes [35]. We observed that treatment with rlipo-E7/CpG enhanced the number of M1-like TAMs and reduced the number of M2-like TAMs (Figure 6 c, d). Interestingly, CD80^+^ TAM numbers were dramatically increased in the rlipo-E7m/CpG group. CD80^+^ TAMs have an M1-like phenotype that produces Th1-biased cytokines for M1 polarization [36,37]. Furthermore, CD80 is a co-stimulatory molecule in antigen-presenting cells that provides the second signal for priming T cells. Switching of tumor-infiltrating macrophages from M2 to M1 has been reported upon use of a combination of CpG ODN and anti-IL-10 antibodies to suppress tumor growth [38]. Here, our data further indicate that rlipo-E7m/CpG significantly increases the number of antigen-specific CD8^+^ T cells during tumor infiltration (Additional file 8: Figure S8). Reduction of the number of TAMs or immunosuppressive cells may not completely eradicate tumor growth; thus, local cytotoxic T lymphocytes are critical for killing tumor cells. The numbers of local CD8^+^ T cells and antigen-specific CD8^+^ T cells were significantly increased in the tumor microenvironment (Additional file 8: Figure S8). We speculate that these infiltrating CD8^+^ T cells might secrete IFN-γ or other cytokines to shift from the M2 to the M1 phenotype. Although we observed that systemic administration of CpG ODN alone did not induce anti-tumor effects or reduce immunosuppressive cell numbers, CpG ODN may amplify the effects of rlipo-E7m and efficiently eliminate large tumors. Therefore, our findings suggest that the induction of CTL responses and the reduction of immunosuppressive cell numbers are critical for eliminating large tumors.

In conclusion, a single administration of recombinant lipoprotein induced strong anti-tumor immunity in the presence of a TLR9 agonist. Anti-tumor immunity resulted from the induction of antigen-specific CD8^+^ T cells and the reduction of immunosuppressive cells in the tumor microenvironment. Currently, we are investigating the potential critical cells and cytokines involved in the local inhibition of immunosuppressive cells.

## Materials and methods

### Animals, cell line and reagents

C57BL/6 mice were purchased from the National Laboratory Animal Center, Taiwan. TLR9-KO mice were purchased from Oriental Bioservice, Inc. (Tokyo, Japan). All experimental mice were maintained in a pathogen-free environment at the Laboratory Animal Center of the National Health Research Institutes (NHRI). The animals were used in compliance with institutional animal health care regulations, and all animal experimental protocols were approved by the NHRI Institutional Animal Care and Use Committee. For experimentally induced neoplasia in mice, the allowable tumor burden and criteria for euthanasia complied with the NCI Frederick ACUC Guidelines (Involving Experimental Neoplasia Proposals in Mice and Rats, 2006). Tumor survival was determined based on 20% weight loss, unexpected moribundity or an inability to obtain food or water.

The TC-1 cell line expressing the HPV-16 E6 and E7 oncoproteins was a kind gift from Dr. T-C. Wu (Johns Hopkins University, USA) [39]. The cells were grown in RPMI-1640 medium supplemented with 10% (v/v) fetal bovine serum, 50 units/mL penicillin/streptomycin, 0.5 mM sodium pyruvate, 20 mM HEPES (Biological industries, Beit Haemek, Israel) and 0.5 μM β-mercaptoethanol at 37°C under 5% CO_2_.

Oligodeoxynucleotide 1668 (CpG-ODN) was purchased from Invitrogen (Grand Island, NY). Its sequence was 5′-TCC ATG ACG TTC CTG ACG TT-3′ with a phosphorothioate backbone. Recombinant mouse GM-CSF and FLT-3 ligand were purchased from PeproTech, and lipopolysaccharide (LPS; *Escherichia coli* endotoxin serotype 055:B5) was purchased from Sigma-Aldrich. Carboxyfluorescein diacetate succinimidyl ester (CFSE) and propidium iodide (PI) were purchased from Invitrogen™. The PE-conjugated HPV16E7_49-57_/MHC I tetramer was purchased from Beckman Coulter, Inc. The antibodies used in this study, with their respective clones in parentheses, were anti-CD16/32 (2.4G2), anti-CD4 (GK1.5), anti-CD8 (53-6.7), anti-F4/80 (BM8), anti-Gr-1 (RB6-8C5), anti-CD11b (M1/70), anti-IFN-γ (XMG1.1), anti-TNF-α (MP6-XT22), anti-IL-10 (JESS-16E3), anti-Foxp3 (FJK-16s) (all purchased from eBioscience®) and anti-CD45 (EM-05) (GeneTex, Inc). The chemotherapy drug cisplatin was purchased from Sigma Aldrich®.

### Generation of dendritic cell subsets

The pDCs were derived from C57BL/6 mouse bone marrow [40]. Briefly, the tibias were removed from 6-12-week-old mice and rinsed in 75% ethanol. The bone marrow cells were then flushed out and passed through a 70-μm nylon cell strainer (BD Falcon) with lymphocyte culture medium (LCM, RPMI-1640 medium supplemented with 10% (v/v) fetal bovine serum, 50 units/mL penicillin/streptomycin, 20 mM HEPES and 0.5 μM β-mercaptoethanol). After centrifugation at 1,200 rpm for 10 minutes, the bone marrow cells were lysed in 3 mL of RBC lysis buffer (BioLegend®) for 3 minutes, and 10 mL of LCM was added to halt the lysis. The cells were again centrifuged at 1,200 rpm for 10 minutes, and the cell supernatant was discarded. The cells were subsequently resuspended in LCM, and 2 × 10^6^ cells were seeded into a 90 × 15 mm Petri dish (α-Plus) with 10 mL of LCM as well as 100 ng/mL of FLT-3 ligand (PeproTech) or 20 ng/ml of GM-CSF (PeproTech). The cells were incubated at 37°C under 5% CO_2_ for 3 days, and another 10 mL of LCM containing 100 ng/mL of FLT-3 ligand or 20 ng/ml of GM-CSF was added to the cell culture plates (day 7, CD11c^+^ cells ~75%). The floating BMDCs or pDCs were harvested on day 6 or day 7, respectively, and 2 × 10^5^ DCs were seeded into a 96-micro-well plate with 0.1 mL of LCM. The stimulating ligand was dissolved in LCM and subsequently added to the DC culture for an additional 24 hours of incubation. For the DC activation analysis, several secretory cytokines in the culture supernatants were detected by ELISA. All assays were performed in duplicate in three independent experiments.

### Immunization and tumor challenge

To evaluate therapeutic anti-tumor effects, TC-1 cells (2 × 10^5^ per mouse) were implanted subcutaneously into the left flanks of naïve C57BL/6 mice 7, 14 or 25 days prior to immunization. The mice were arbitrarily assigned to groups (6 per group) and were immunized subcutaneously in the dorsum with the indicated doses of rlipo-E7m [19], either alone or as an admixture with 10 μg of CpG ODN, in a total volume of 100 μL in PBS for each mouse. To monitor tumor growth, the tumors were measured with electronic calipers three times weekly. The tumor volume was calculated using the formula length x width^2^ × 1/2.

TC-1 cancer cells (2 × 10^5^) were inoculated into C57BL/6 mice by intravenous injection to establish an experimental animal model of metastatic lung cancer [41]. After 14 days, a single dose of PBS, rlipo-E7m, CpG or rlipo-E7m/CpG was subcutaneously injected into the mice to evaluate the therapeutic effects of these compounds.

### ELISPOT assay

The IFN-γ ELISPOT assay was performed according to the manufacturer’s instructions (eBioscience). Briefly, the ELISPOT plate (MSIP, Millipore) was pre-coated with anti-mouse IFN-γ capture antibody (AN18) overnight and subsequently blocked with LCM at room temperature for 2 hours. Splenocytes (1 × 10^6^ per well) were plated in duplicate along with an H2-D^b^-restricted CTL epitope (HPV16E7_49-57_) at a concentration of 10 μg/mL or with control peptides at 37°C for 48 hours. Following incubation, the plates were washed and incubated with biotinylated anti-mouse IFN-γ detection antibody (R46A2) for 2 hours, followed by incubation with avidin-HRP for 30 minutes and color development with AEC substrate reagents according to the manufacturer’s instructions (Sigma).

### Flow cytometry

To characterize the populations of myeloid-derived suppressor cells (MDSCs), regulatory T cells (Tregs) and tumor-associated macrophages in immunized mice, RBC-lysed splenocytes or tumor cells derived from tumor-free and/or tumor-bearing mice were incubated with an anti-CD16/CD32 antibody to block non-specific binding and were subsequently stained with fluorochrome-conjugated monoclonal antibodies for surface marker and intracellular staining according to the manufacturer’s instructions (eBioscience®). Briefly, the cell suspension was stained with FITC-conjugated anti-CD4, PE-conjugated anti-CD25 and PE-Cy5-conjugated anti-Foxp3 to quantify Tregs; PE-conjugated anti-CD11b and PE-Cy7-conjugated anti-Gr-1 to quantify MDSCs; FITC-conjugated anti-F4/80 and PE-conjugated anti-CD11b to quantify TAMs and APC-conjugated anti-CD45 to quantify tumor-infiltrating leukocytes. For the intracellular detection of IFN-γ-secreting CD8^+^ T cells, RBC-lysed splenocytes derived from immunized C57BL/6 mice were re-stimulated with 10 μg/mL of H2-D^b^-restricted CTL epitopes (HPV16 E7_49-57_) for 48 hours. The cells were subsequently harvested and stained with FITC-conjugated anti-CD8 and PE-conjugated anti-IFN-γ using a standard intracellular protocol. The E7-specific CD8^+^ T cells were stained with a PE-conjugated H-2D^b^/RAH tetramer and FITC-conjugated anti-CD8. The percentage of H-2D^b^/RAH^+^ CD8^+^ T cells was determined by flow cytometry (FACSCalibur, BD Bioscience, San Jose, CA). All of the data were analyzed using a FACSCalibur flow cytometer and the CellQuest software. All analyses were conducted on a gated lymphocyte population.

### *In vivo* cytolytic assay

To detect antigen-specific cytolytic activity in the immunized mice *in vivo*, specific or irrelevant peptide-pulsed syngeneic splenocytes were used as target cells in a killing assay. The RBC-lysed splenocytes were counted and divided into two equal portions. These two portions were incubated at a density of 2 × 10^7^ cells/ml with a specific peptide (HPV16 E7_49-57_) and a non-specific peptide (OVA_257-264_) at 37°C for 30 minutes. To differentiate between the peptide-pulsed target cells, the two subsets of cells were labeled with CFSE^hi^ (5 μM) and CFSE^lo^ (0.5 μM) at 37°C for 10 minutes. Both samples of cells were resuspended at 2 × 10^7^ cells/ml and subsequently mixed at a 1:1 ratio (1 × 10^7^:1 × 10^7^) in PBS prior to adoptive transfer into immunized mice via tail vein injection 7 days after the previous immunization. The experimental cells were harvested 18 hours after adoptive transfer and analyzed using a FACSCalibur flow cytometer (BD Bioscience). The percentage of specific lysis was calculated using the following equation: % Specific lysis = [1-(%CFSE ^hi^/%CFSE ^lo^)] × 100.

### Depletion of leukocyte subpopulations *in vivo*

CD4^+^, CD8^+^ or NK cells were depleted *in vivo* using 0.5 mg of anti-CD4 (GK1.5, eBioscience®), anti-CD8 (53-6.7, eBioscience®) or anti-NK1.1 (PK136, BioLegend®) injected intraperitoneally into mice one day prior to immunization. Rat IgG or mouse IgG (0.5 mg) (Invitrogen™) was used as the control antibody. The depletion efficiency was ~90% as determined by flow cytometry. Mice were implanted with TC-1 cells (2 × 10^5^ per mouse) subcutaneously. Seven days later, 10 μg of rlipo-E7m/CpG was injected s.c. into the dorsum in a total volume of 100 μL. To monitor tumor growth, the tumors were measured with electronic calipers three times weekly. The tumor volume was calculated using the formula length × width^2^ × 1/2.

### Statistical analysis

Statistical analyses were performed using Prism version 5.02 (GraphPad, CA, USA). Kaplan-Meier analysis was performed on the survival rates of the mice. The statistical significance of the differences between the groups was assessed using a two-tailed Student’s *t* test. For all results, *P* < 0.05 was considered statistically significant.

## Abbreviations

TLR: Toll-like receptor; CTL: Cytotoxic T lymphocyte; CpG ODN: CpG oligodeoxynucleotides; DC: Dendritic cell; pDC: Plasmacytoid dendritic cell; MDSC: Myeloid-derived suppressor cell; Treg: Regulatory T cell; TAM: Tumor-associated macrophage.

## Competing interests

The authors declare no financial or commercial conflicts of interest.

## Authors’ contributions

LSC, YCY and CCW performed the experiments with contributions from CHL, and HWC analyzed the data. HMH and SJL designed the experiments and wrote the manuscript. All authors read and approved the final manuscript.

## Supplementary Material

Additional file 1: Figure S1 rlipo-E7m induced long-lasting anti-tumor effects in the presence of CpG ODN. A total of 2 × 10^5^ TC-l tumor cells were *s.c.* implanted into C57BL/6 mice. At 7 days post-tumor cell implantation, tumor-bearing mice were administered a single dose of **(a)** PBS or **(b)** rlipo-E7m (10 μg/ mouse) + CpG via *s.c*. injection. The data represent the individual tumor volume over time following tumor cell implantation. All tumors were measured at regular intervals using electronic calipers The mice were euthanized when the tumor diameter reached 20 mm or when necessary due to the moribund status of the animals. The numbers of mice in each group are indicated in each graph. The tumor volume was calculated using the formula length × width × width/2 (mm^3^). Click here for file

Additional file 2: Figure S2 Induction of CTL responses in tumor-bearing mice. **(a)** CFSE-labeled cells were *i.v.* injected into tumor-bearing mice (*n*=6 per group) 10 days after immunization. After 18 h, spleen cells were isolated from immunized mice and analyzed via flow cytometry. **(b)** The tumor cells were stained with an RAH/MHC I tetramer and anti-CD8 antibodies. The data represent the percentage of CD8^+^RAH Tet^+^ in all cells. *P < 0.05, **P < 0.01, ***P < 0.001. Click here for file

Additional file 3: Figure S3 rlipo-E7m induced long-lasting memory of anti-tumor effects. After treatment with rlipo-E7m/CpG, the tumor-free mice (*n*=9) were re-challenged with TC-L cells (2 × 105/mouse) subcutaneously at 135 days (naive mice were used as a control). Kaplan-Meier analysis was performed on the mice survival data (***P < 0.001, naive versus rlipo-E7m/CpG). Tumor survival was determined based on 20% weight loss, unexpected moribundity or an inability to obtain food or water (as described in Materials and Methods). Click here for file

Additional file 4: Figure S4 rhpo-F7m- and CpG ODN-mediated therapeutic effects are abolished in thymoma tumor-bearing mice. A total of 5 × 10^4^ EL4 tumor cells were subcutaneously (*s.c*.) implanted into C57BL/6 (*n*=5 per group) mice. Seven days after tumor cell implantation, the tumor-hearing mice were administered a single dose of PBS, rlipo-E7m or rlipo-F7m + CpG via *s.c*. injection The data represent the mean tumor volume at 17, 24 and 27 days after tumor cell implantation (means + SD). All the tumor sizes were measured using electronic calipers and calculated using the formula length × width × width/2 (mm^3^). Click here for file

Additional file 5: Figure S5 The combination of the recombinant lipoprotein and CpG ODN shows no synergistic effects on BMDCs activation. Mouse bone marrow-derived BMDCs at 6 days after GM-CSF supplementation were used to the evaluate synergistic effects of innate immune cell activation induced through rlipo-E7m in combi nation with CpG ODN, The BMDC supernatants were collected for cytokine detection at 24 h after stimulation. The cytokines IL-l2p7O, TNF- α, IL-6 and IL-10 were analyzed through ELISA to assess the extent of DCs activation. The presented data represent the mean + SD of duplicate BMDCs cultures from three independent experiments. Click here for file

Additional file 6: Figure S6 Expression of TLR2 and TLR in dendritic cell subsets. Total RNA of BMDCs, pDCs or splenic DCs were extracted using the total RNA isolation kit. The obtained cDNA was diluted 1/25 with water and 10 μL were used for amplification. The PCR was performed with the SYBRR Green PCR Master Mix. Gene expression of TLR 2 and TLR 9 was determined by quantitative real-time RT-PCR and normalized to GAPDH. Click here for file

Additional file 7: Figure S7 TLR9 agonist CpG enhanced anti-tumor effects and antigen presentation of recombinant lipoimmunogen. **(a)** BMTCs or pDCs were pulsed with rlipo-OVA( 100 nM) in the presence or absence of CpG (100 nM) for 18 hr. Purity > 90% The CD8^+^ cells isolated from OT-l mice (purity > 90%) were cultured with protein-plused DCs in the ratio 5:1 for 72 hr. T cell proliferation was determined by [^3^H] -thymidine incorporation. **(b)** C57BL/6 mice (*n*=6 per group) were inoculated with 2 ×l0^4^ of EG7 cells in a total volume 200 μ1 subcutaneously. After 3 days, 10 μg of rOVA, rlipo-OVA, rlipo-OVA/CpG or PBS was injected *s.c*. Tumor growth was observed three times per week. The tumor volume was shown as length × width × width/2 (mm^3^). Data are expressed as mean SEM. Click here for file

Additional file 8: Figure S8 rlipo-E7m immunization leads to increased numbers of tumor-infiltrating antigen-specific CD8^+^ T cells in the presence of CpG ODN. Tumor-bearing mice (*n*=6 per group) were immunized with rlipo-E7m (10 μg/mouse), rlipo-E7m (10 μg/mouse) + CpG (10 μg/mouse) or PBS as a control at 14 days post-tumor cell implantation. The tumors removed from tumor-bearing mice at 24 days post-tumor implantation were minced and crushed through a 70-μm filter, and the total cells were stained with antibodies against the indicated markers 50,000 events were acquired for each sample The data represent the percentages of **(a)** total CD8^+^ cells and **(b)** E7-specific CD8^+^ cells among tumor-infiltrating CD45^+^ cells. Significant differences are indicated by the *P* values in the graph. Click here for file
