# Supplementary material for: Toll-like receptor 9 agonist enhances anti-tumor immunity and inhibits tumor-associated immunosuppressive cells numbers in a mouse cervical cancer model following recombinant lipoprotein therapy
Source: Mol Cancer. 2014 Mar 19;13:60. doi: 10.1186/1476-4598-13-60 (PMC4000133; doi:10.1186/1476-4598-13-60)
Supplement: Additional file 1: Figure S1 — rlipo-E7m induced long-lasting anti-tumor effects in the presence of CpG ODN. A total of 2 × 10^5^ TC-l tumor cells were s.c. implanted into C57BL/6 mice. At 7 days post-tumor cell implantation, tumor-bearing mice were administered a single dose of (a) PBS or (b) rlipo-E7m (10 μg/ mouse) + CpG via s.c. injection. The data represent the individual tumor volume over time following tumor cell implantation. All tumors were measured at regular intervals using electronic calipers The mice were euthanized when the tumor diameter reached 20 mm or when necessary due to the moribund status of the animals. The numbers of mice in each group are indicated in each graph. The tumor volume was calculated using the formula length × width × width/2 (mm3). [file 1476-4598-13-60-S1.pdf]

Additional file 1

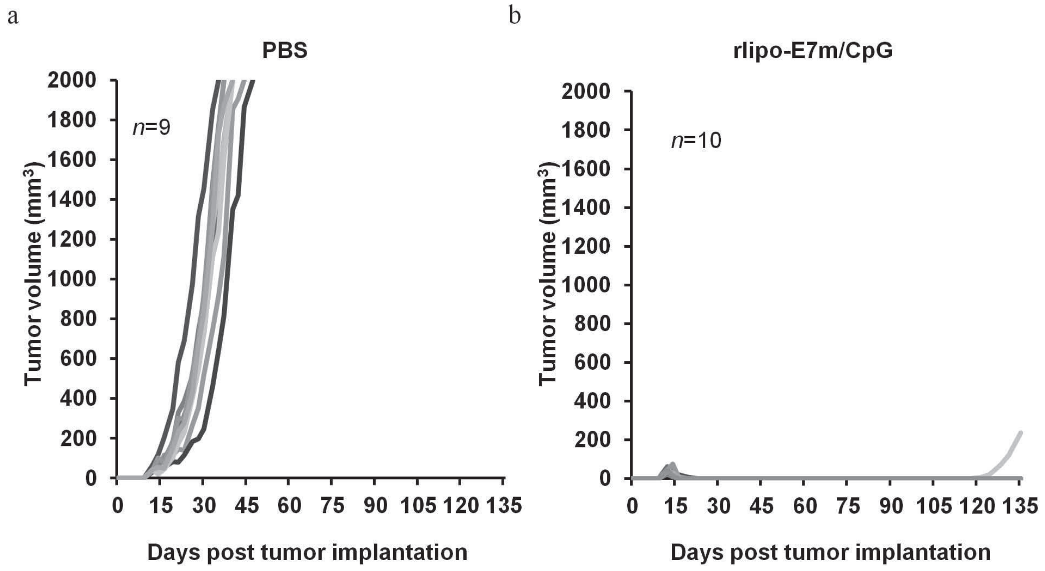

Figure S1: rlipo-E7m induced long-lasting anti-tumor effects in the presence of CpG ODN. A total of 2 x 10^5 TC-1 tumor cells were *s.c.* implanted into C57BL/6 mice. At 7 days post-tumor cell implantation, tumor-bearing mice were administered a single dose of (a) PBS or (b) rlipo-E7m (10 µg/mouse) + CpG via *s.c.* injection. The data represent the individual tumor volume over time following tumor cell implantation. All tumors were measured at regular intervals using electronic calipers. The mice were euthanized when the tumor diameter reached 20 mm or when necessary due to the moribund status of the animals. The numbers of mice in each group are indicated in each graph. The tumor volume was calculated using the formula length × width × width/2 (mm^3).
