# Supplementary material for: Toll-like receptor 9 agonist enhances anti-tumor immunity and inhibits tumor-associated immunosuppressive cells numbers in a mouse cervical cancer model following recombinant lipoprotein therapy
Source: Mol Cancer. 2014 Mar 19;13:60. doi: 10.1186/1476-4598-13-60 (PMC4000133; doi:10.1186/1476-4598-13-60)
Supplement: Additional file 2: Figure S2 — Induction of CTL responses in tumor-bearing mice. (a) CFSE-labeled cells were i.v. injected into tumor-bearing mice (n=6 per group) 10 days after immunization. After 18 h, spleen cells were isolated from immunized mice and analyzed via flow cytometry. (b) The tumor cells were stained with an RAH/MHC I tetramer and anti-CD8 antibodies. The data represent the percentage of CD8+RAH Tet+ in all cells. *P < 0.05, **P < 0.01, ***P < 0.001. [file 1476-4598-13-60-S2.pdf]

Additional file 2

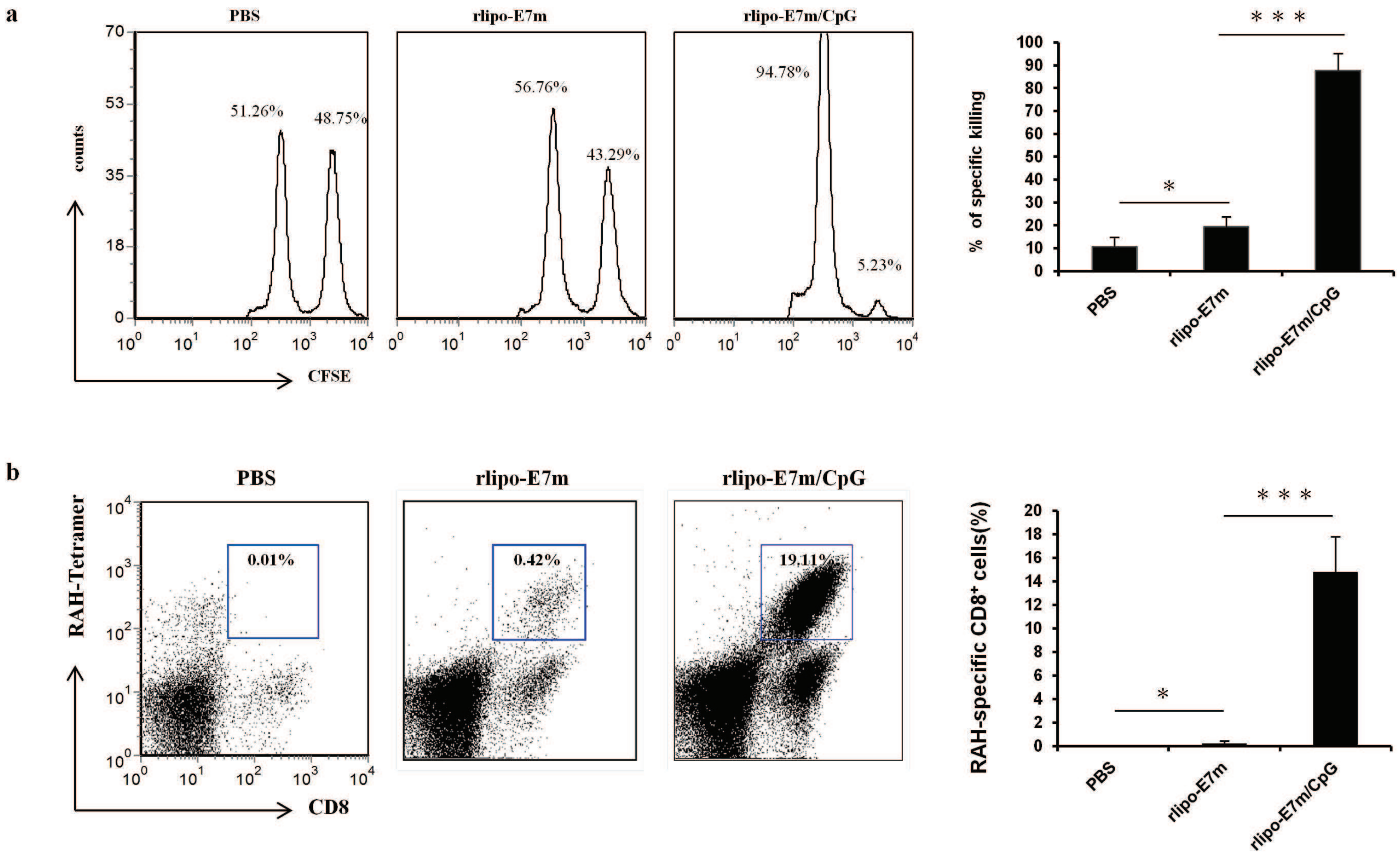

Figure S2: Induction of CTL responses in tumor-bearing mice. (a) CFSE-labeled cells were *i.v.* injected into tumor-bearing mice (*n*=6 per group) 10 days after immunization. After 18 h, spleen cells were isolated from immunized mice and analyzed via flow cytometry. (b) The tumor cells were stained with an RAH/MHC I tetramer and anti-CD8 antibodies.The data represent the percentage of $CD8^{+}$RAH Tet^{+} in all cells. * $P < 0.05$, ** $P < 0.01$, *** $P < 0.001$.
