# Supplementary material for: Toll-like receptor 9 agonist enhances anti-tumor immunity and inhibits tumor-associated immunosuppressive cells numbers in a mouse cervical cancer model following recombinant lipoprotein therapy
Source: Mol Cancer. 2014 Mar 19;13:60. doi: 10.1186/1476-4598-13-60 (PMC4000133; doi:10.1186/1476-4598-13-60)
Supplement: Additional file 3: Figure S3 — rlipo-E7m induced long-lasting memory of anti-tumor effects. After treatment with rlipo-E7m/CpG, the tumor-free mice (n=9) were re-challenged with TC-L cells (2 × 10^5^/mouse) subcutaneously at 135 days (naive mice were used as a control). Kaplan-Meier analysis was performed on the mice survival data (***P < 0.001, naive versus rlipo-E7m/CpG). Tumor survival was determined based on 20% weight loss, unexpected moribundity or an inability to obtain food or water (as described in Materials and Methods). [file 1476-4598-13-60-S3.pdf]

Additional file 3

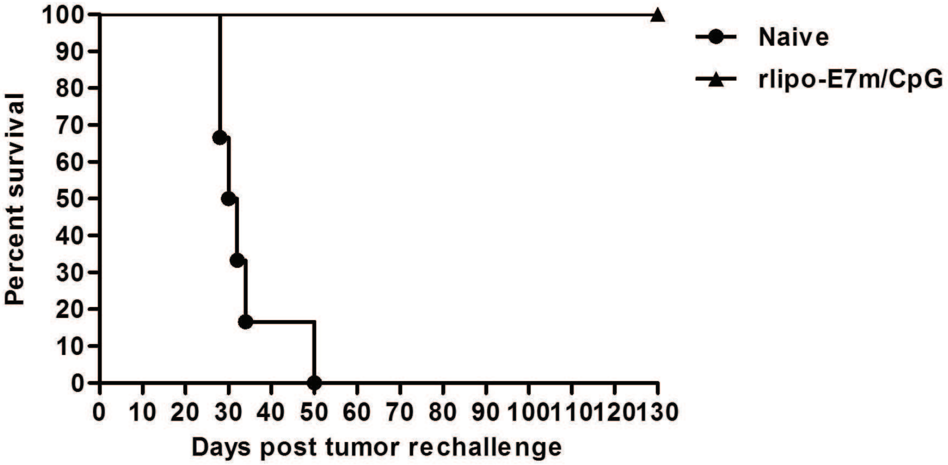

Figure 3S: rlipo-E7m induced long-lasting memory of anti-tumor effects. After treatment with rlipo-E7m/CpG, the tumor-free mice (*n*=9) were re-challenged with TC-1 cells (2 x 10^5/mouse) subcutaneously at 135 days (naive mice were used as a control). Kaplan-Meier analysis was performed on the mice survival data (*** $P < 0.001$, naive versus rlipo-E7m/CpG). Tumor survival was determined based on 20% weight loss, unexpected moribundity or an inability to obtain food or water (as described in Materials and Methods).
