# Supplementary material for: Toll-like receptor 9 agonist enhances anti-tumor immunity and inhibits tumor-associated immunosuppressive cells numbers in a mouse cervical cancer model following recombinant lipoprotein therapy
Source: Mol Cancer. 2014 Mar 19;13:60. doi: 10.1186/1476-4598-13-60 (PMC4000133; doi:10.1186/1476-4598-13-60)
Supplement: Additional file 4: Figure S4 — rhpo-F7m- and CpG ODN-mediated therapeutic effects are abolished in thymoma tumor-bearing mice. A total of 5 × 10^4^ EL4 tumor cells were subcutaneously (s.c.) implanted into C57BL/6 (n=5 per group) mice. Seven days after tumor cell implantation, the tumor-hearing mice were administered a single dose of PBS, rlipo-E7m or rlipo-F7m + CpG via s.c. injection The data represent the mean tumor volume at 17, 24 and 27 days after tumor cell implantation (means + SD). All the tumor sizes were measured using electronic calipers and calculated using the formula length × width × width/2 (mm3). [file 1476-4598-13-60-S4.pdf]

Additional file 4

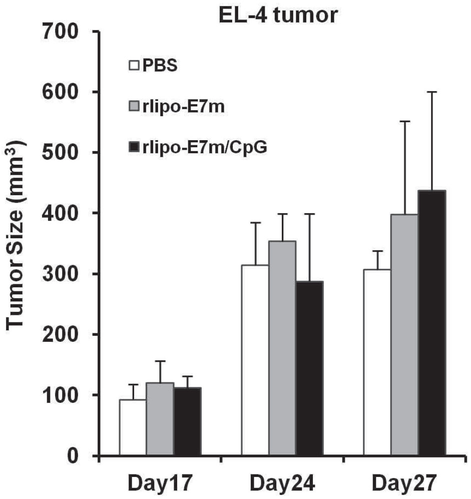

Figure S4: rlipo-E7m- and CpG ODN-mediated therapeutic effects are abolished in thymoma tumor-bearing mice. A total of 5 x 10^4 EL4 tumor cells were subcutaneously (*s.c.*) implanted into C57BL/6 (*n*=5 per group) mice. Seven days after tumor cell implantation, the tumor-bearing mice were administered a single dose of PBS, rlipo-E7m or rlipo-E7m + CpG via *s.c.* injection. The data represent the mean tumor volume at 17, 24 and 27 days after tumor cell implantation (means + SD). All the tumor sizes were measured using electronic calipers and calculated using the formula length $\times$ width $\times$ width /2 (mm^3).
