# Supplementary material for: Toll-like receptor 9 agonist enhances anti-tumor immunity and inhibits tumor-associated immunosuppressive cells numbers in a mouse cervical cancer model following recombinant lipoprotein therapy
Source: Mol Cancer. 2014 Mar 19;13:60. doi: 10.1186/1476-4598-13-60 (PMC4000133; doi:10.1186/1476-4598-13-60)
Supplement: Additional file 5: Figure S5 — The combination of the recombinant lipoprotein and CpG ODN shows no synergistic effects on BMDCs activation. Mouse bone marrow-derived BMDCs at 6 days after GM-CSF supplementation were used to the evaluate synergistic effects of innate immune cell activation induced through rlipo-E7m in combi nation with CpG ODN, The BMDC supernatants were collected for cytokine detection at 24 h after stimulation. The cytokines IL-l2p7O, TNF- α, IL-6 and IL-10 were analyzed through ELISA to assess the extent of DCs activation. The presented data represent the mean + SD of duplicate BMDCs cultures from three independent experiments. [file 1476-4598-13-60-S5.pdf]

Additional file 5

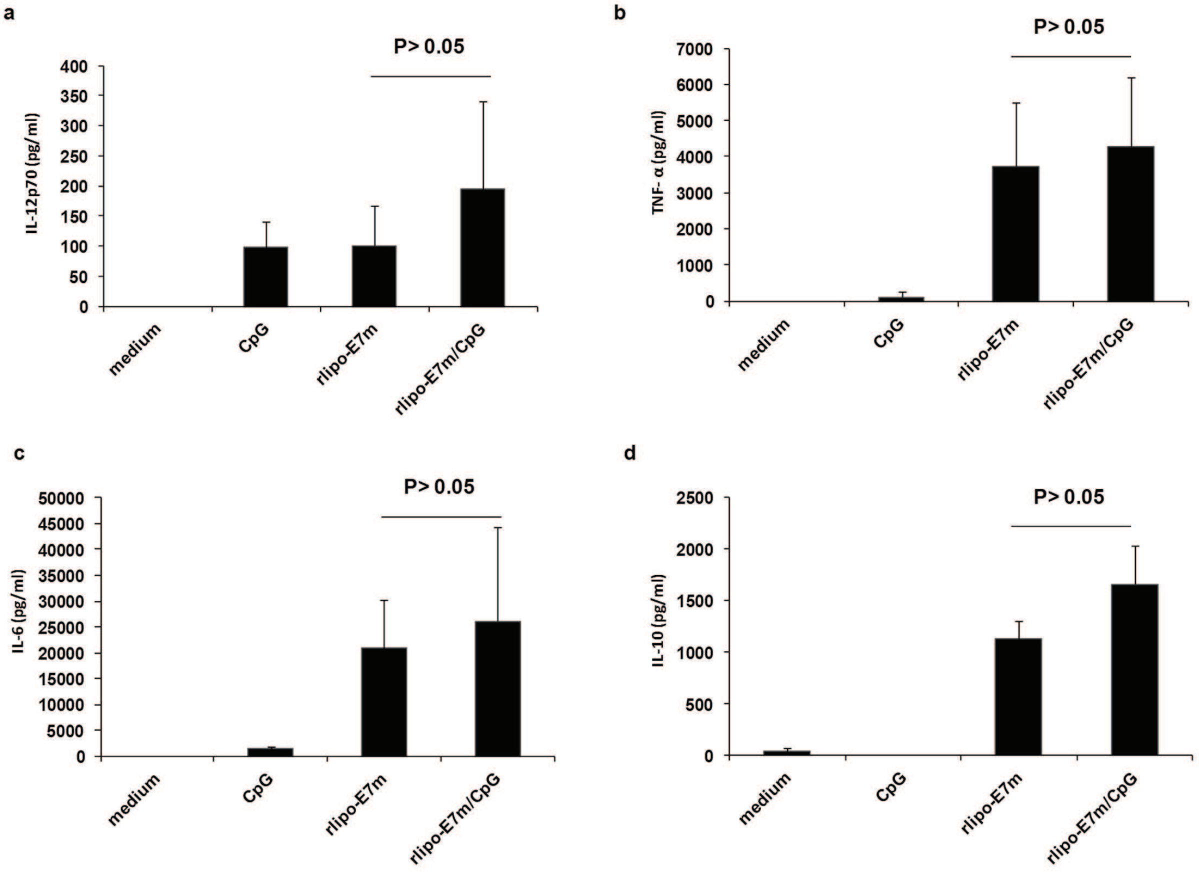

Figure S5: The combination of the recombinant lipoprotein and CpG ODN shows no synergistic effects on BMDCs activation. Mouse bone marrow-derived BMDCs at 6 days after GM-CSF supplementation were used to the evaluate synergistic effects of innate immune cell activation induced through rlipo-E7m in combination with CpG ODN. The BMDC supernatants were collected for cytokine detection at 24 h after stimulation. The cytokines IL-12p70, TNF- α, IL-6 and IL-10 were analyzed through ELISA to assess the extent of DCs activation. The presented data represent the mean + SD of duplicate BMDCs cultures from three independent experiments.
