# Supplementary material for: Toll-like receptor 9 agonist enhances anti-tumor immunity and inhibits tumor-associated immunosuppressive cells numbers in a mouse cervical cancer model following recombinant lipoprotein therapy
Source: Mol Cancer. 2014 Mar 19;13:60. doi: 10.1186/1476-4598-13-60 (PMC4000133; doi:10.1186/1476-4598-13-60)
Supplement: Additional file 6: Figure S6 — Expression of TLR2 and TLR in dendritic cell subsets. Total RNA of BMDCs, pDCs or splenic DCs were extracted using the total RNA isolation kit. The obtained cDNA was diluted 1/25 with water and 10 μL were used for amplification. The PCR was performed with the SYBRR Green PCR Master Mix. Gene expression of TLR 2 and TLR 9 was determined by quantitative real-time RT-PCR and normalized to GAPDH. [file 1476-4598-13-60-S6.pdf]

Additional file 6

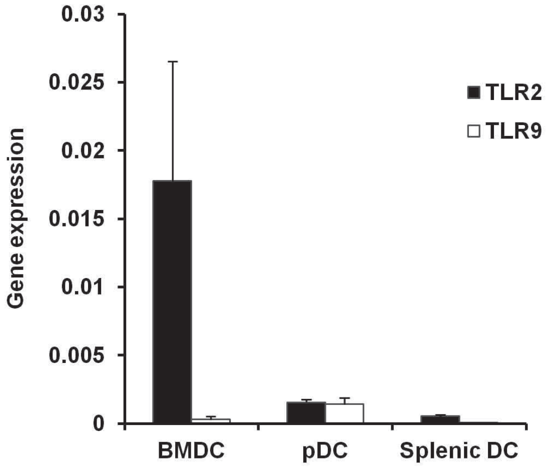

Figure S6: Expression of TLR2 and TLR in dendritic cell subsets. Total RNA of BMDCs, pDCs or splenic DCs were extracted using the total RNA isolation kit. The obtained cDNA was diluted 1/25 with water and 10 μL were used for amplification. The PCR was performed with the SYBRR Green PCR Master Mix. Gene expression of TLR 2 and TLR 9 was determined by quantitative real-time RT-PCR and normalized to GAPDH.
