# Supplementary material for: Toll-like receptor 9 agonist enhances anti-tumor immunity and inhibits tumor-associated immunosuppressive cells numbers in a mouse cervical cancer model following recombinant lipoprotein therapy
Source: Mol Cancer. 2014 Mar 19;13:60. doi: 10.1186/1476-4598-13-60 (PMC4000133; doi:10.1186/1476-4598-13-60)
Supplement: Additional file 7: Figure S7 — TLR9 agonist CpG enhanced anti-tumor effects and antigen presentation of recombinant lipoimmunogen. (a) BMTCs or pDCs were pulsed with rlipo-OVA( 100 nM) in the presence or absence of CpG (100 nM) for 18 hr. Purity > 90% The CD8+ cells isolated from OT-l mice (purity > 90%) were cultured with protein-plused DCs in the ratio 5:1 for 72 hr. T cell proliferation was determined by [3H] -thymidine incorporation. (b) C57BL/6 mice (n=6 per group) were inoculated with 2 ×l04 of EG7 cells in a total volume 200 μ1 subcutaneously. After 3 days, 10 μg of rOVA, rlipo-OVA, rlipo-OVA/CpG or PBS was injected s.c. Tumor growth was observed three times per week. The tumor volume was shown as length × width × width/2 (mm3). Data are expressed as mean SEM. [file 1476-4598-13-60-S7.pdf]

Additional file 7

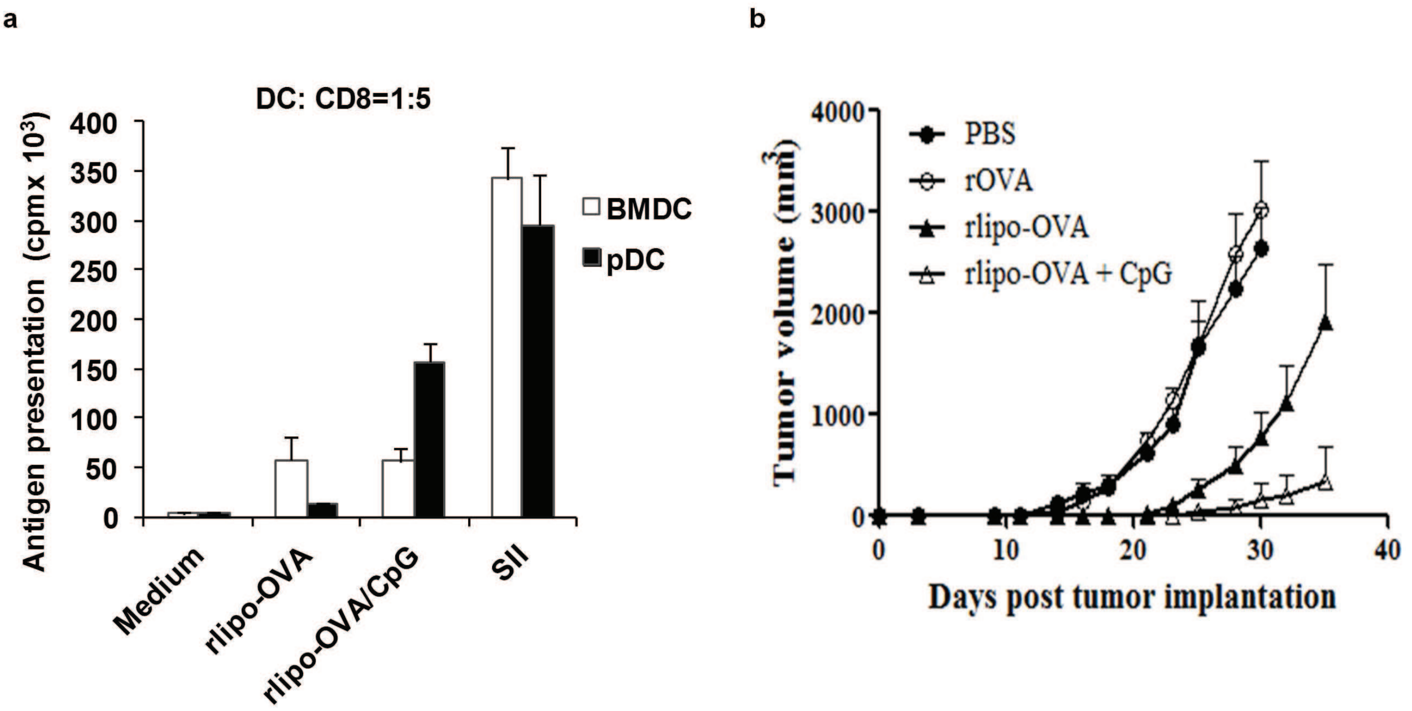

Figure S7: TLR9 agonist CpG enhanced anti-tumor ffects and antigen presentation of recombinant lipoimmunogen. (a) BMDCs or pDCs were pulsed with rlipo-OVA(100 nM) in the presence or absence of CpG (100 nM) for 18 hr. Purity＞90% The $CD8^+$ cells isolated from OT-1 mice (purity > 90 %) were cultured with protein-plused DCs in the ratio 5:1 for 72 hr. T cell proliferation was determined by $[^3H]$ -thymidine incorporation. (b) C57BL/6 mice (*n*=6 per group) were inoculated with 2 $x10^4$ of EG7 cells in a total volumne 200 µl subcutaneously. After 3 days, 10 µg of rOVA , rlipo-OVA , rlipo-OVA/CpG or PBS was injected s.c. Tumor growth was observed three times per week. The tumor volume was shown as length x width x width/2 (mm^3). Data are expressed as mean ＋ SEM.
