# Supplementary material for: Toll-like receptor 9 agonist enhances anti-tumor immunity and inhibits tumor-associated immunosuppressive cells numbers in a mouse cervical cancer model following recombinant lipoprotein therapy
Source: Mol Cancer. 2014 Mar 19;13:60. doi: 10.1186/1476-4598-13-60 (PMC4000133; doi:10.1186/1476-4598-13-60)
Supplement: Additional file 8: Figure S8 — rlipo-E7m immunization leads to increased numbers of tumor-infiltrating antigen-specific CD8+ T cells in the presence of CpG ODN. Tumor-bearing mice (n=6 per group) were immunized with rlipo-E7m (10 μg/mouse), rlipo-E7m (10 μg/mouse) + CpG (10 μg/mouse) or PBS as a control at 14 days post-tumor cell implantation. The tumors removed from tumor-bearing mice at 24 days post-tumor implantation were minced and crushed through a 70-μm filter, and the total cells were stained with antibodies against the indicated markers 50,000 events were acquired for each sample The data represent the percentages of (a) total CD8+ cells and (b) E7-specific CD8+ cells among tumor-infiltrating CD45+ cells. Significant differences are indicated by the P values in the graph. [file 1476-4598-13-60-S8.pdf]

Additional file 8

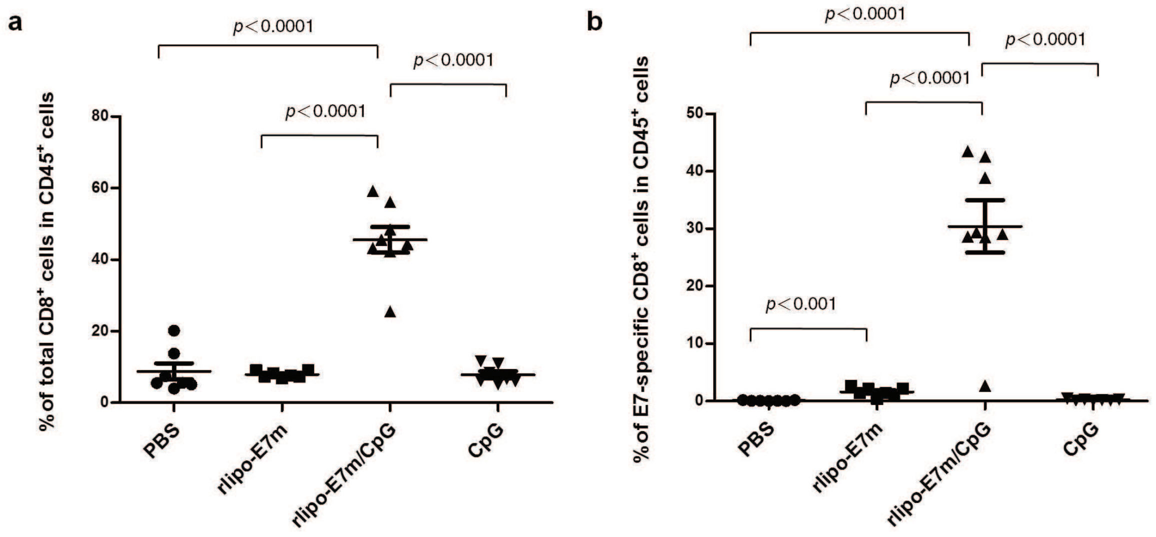

Figure S8: rlipo-E7m immunization leads to increased numbers of tumor-infiltrating antigen-specific CD8$^+$ T cells in the presence of CpG ODN. Tumor-bearing mice (n=6 per group) were immunized with rlipo-E7m (10 µg/mouse), rlipo-E7m (10 µg/mouse) + CpG (10 µg/mouse) or PBS as a control at 14 days post-tumor cell implantation. The tumors removed from tumor-bearing mice at 24 days post-tumor implantation were minced and crushed through a 70-µm filter, and the total cells were stained with antibodies against the indicated markers; 50,000 events were acquired for each sample. The data represent the percentages of (a) total CD8$^+$ cells and (b) E7-specific CD8$^+$ cells among tumor-infiltrating CD45$^+$ cells. Significant differences are indicated by the P values in the graph.
